# A health promotion model approach in exploring self-management and glycemic control in type 2 diabetes: the moderating effects of self-efficacy and social support

**DOI:** 10.3389/fcdhc.2025.1573805

**Published:** 2025-06-11

**Authors:** Sylvi Ndatila Amunkete, Elihuruma Eliufoo Stephano, Mtoro J. Mtoro, Feng Hui

**Affiliations:** ^1^ Community Health Department, Xiangya School of Nursing, Central South University, Changsha, China; ^2^ Department of Clinical Teaching and Research, Second Xiangya Hospital, Central South University, Changsha, China; ^3^ TILAM International, Dar es Salaam, Tanzania

**Keywords:** diabetes management, fasting blood glucose (FBG), health promotion model, self-efficacy, self-management practices, self-monitoring of blood glucose, social support

## Abstract

**Background:**

As the prevalence of diabetes and its related complications continues to rise, understanding the factors that influence glycemic control is crucial for improving patient outcomes. This study aimed to explore the roles of self-management, social support, and self-efficacy in moderating fasting blood glucose (FBG) levels in individuals with type 2 diabetes mellitus (T2DM).

**Methods:**

A health facility-based cross-sectional study was conducted in Windhoek, Namibia with a sample size of 315 T2DM patients receiving follow-up care. Descriptive statistics and Pearson correlation analysis were conducted to examine the relationship between self-management and FBG. Linear regression and moderation analyses were used to determine the moderating effects.

**Results:**

The study revealed 34.3% engaged in self-monitoring of FBG, while medication adherence was high at an average of 7 days. A significant negative correlation between self-management practices and FBG levels was identified (r = -0.349, p < 0.028). Self-management, self-efficacy, and social support accounted for 43.1% of FBG variation, with self-management emerging as a significant predictor (β = -0.903, p < 0.001). Additionally, social support and self-efficacy significantly moderated the relationship between self-management and FBG levels.

**Conclusion:**

This study showed the significant moderating roles of social support and self-efficacy in the relationship between self-management practices and FBG levels in patients with diabetes. These results highlight the importance of comprehensive diabetes management programs focusing on individual behavioral changes, enhancing social support networks, and boosting self-efficacy.

## Background

The increasing prevalence of diabetes, particularly Type 2 diabetes mellitus (T2DM), represents a significant public health challenge globally, with rising concerns regarding management and health outcomes (1). The World Health Organization (WHO) estimates that approximately 422 million individuals live with diabetes, predominantly in low- and middle-income countries (2). Globally, T2DM represents about 96% of all diabetes cases, fuelled by factors such as high body mass index (BMI), sedentary lifestyles, and poor dietary habits, which have been implicated as primary contributors to its onset (1, 3). The trend is alarming, particularly given that diabetes is associated with numerous complications, including cardiovascular diseases and renal failure, leading to increased morbidity and mortality (4). Effective management strategies focusing on self-management behaviors have become crucial (1, 4).

Self-management refers to the ability of patients to regulate their health and manage their conditions effectively through adherence to medication regimens, physical activity, and dietary restrictions (5). However, studies reveal that many individuals with T2DM do not achieve optimal fasting blood glucose control due to various barriers, underscoring the need for comprehensive intervention strategies (6, 7).

In Sub-Saharan Africa (SSA), diabetes poses a triple challenge, which includes epidemic growth, insufficient healthcare resources, and overlapping health issues such as infectious diseases (8). The prevalence of T2DM in SSA has seen rapid growth and is expected to rise from approximately 15.5 million in 2017 to 40.7 million by 2045 (9–11). Namibia, in particular, is experiencing a rising trend in diabetes cases, estimated at a prevalence of 7.3% in 2021, with projections suggesting a further increase to 7.9% by 2030 (12). The Diabetes Atlas estimated that diabetes accounted for about 4% of deaths in Namibia, highlighting urgent intervention needs for patient education and management support (12, 13). Despite the alarming statistics, numerous barriers hinder effective self-management and healthcare access in Namibia. These challenges include inadequate healthcare facilities, long waiting times, limited access to diabetes education resources, and insufficient support systems.

**Figure 1:**
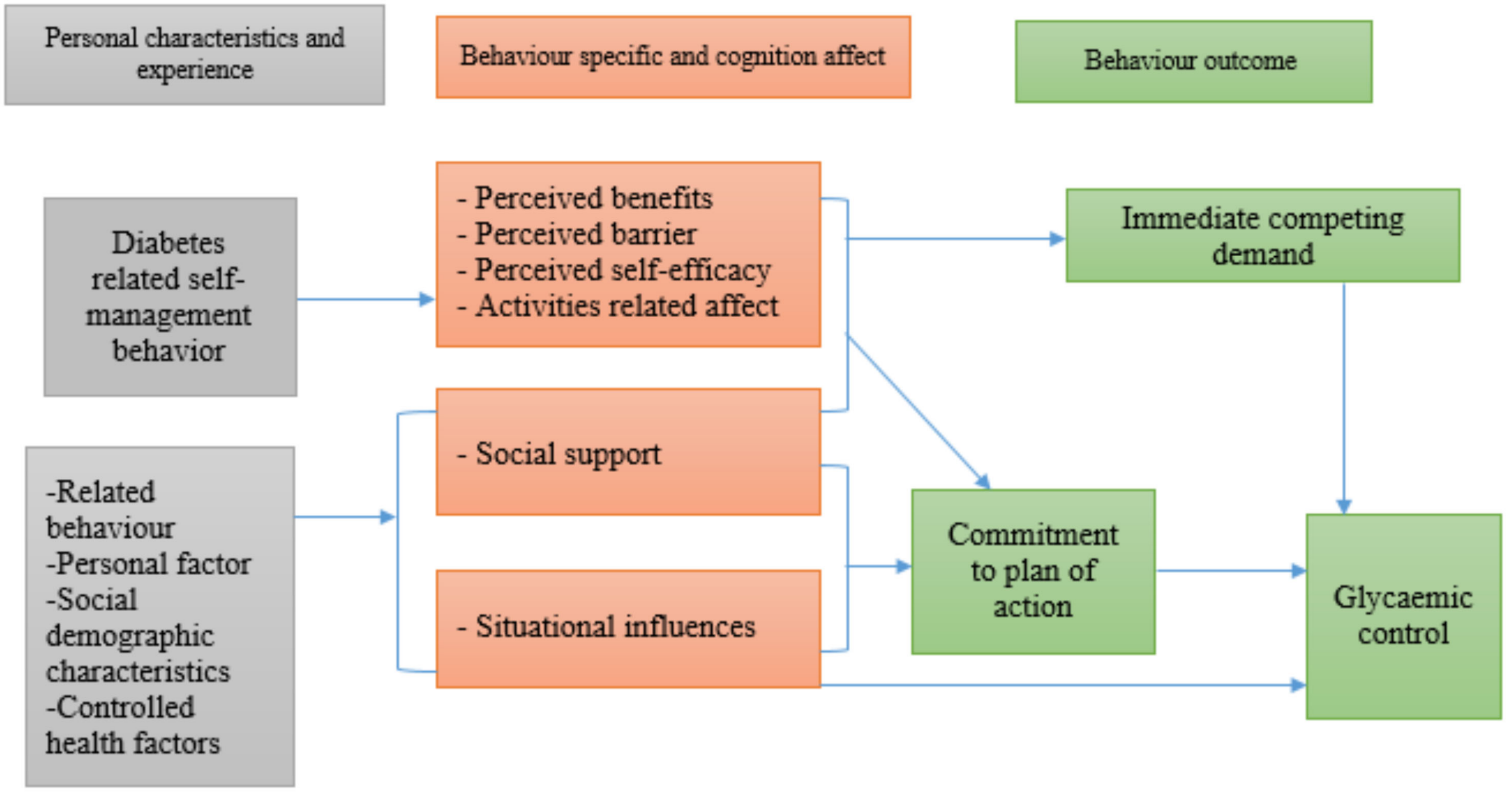
Conceptual framework of the relationship between self-management and the moderation effects of social support and self-efficacy showing the interactive nature of diabetes self-management, illustrating how social support and self-efficacy independently and jointly moderate the pathway between self-management behaviors and favorable health outcomes in T2DM patients.

This study employs Nola Pender’s Health Promotion Model (HPM) to explore the relationship between self-management and fasting blood glucose among Type 2 diabetic patients in Windhoek, Namibia (14). The HPM emphasizes the multifaceted nature of health behaviors and interventions that prompt self-management, which is highly relevant in diabetes care. Notably, the model incorporates the dual roles of self-efficacy and social support as moderating factors that can enhance self-management behaviors and improve health outcomes. Research indicates that self-efficacy, an individual’s belief in their ability to perform tasks associated with managing their health, can directly influence treatment adherence and self-management effectiveness (15). Moreover, social support has been reported to buffer the impacts of diabetes distress, substantially contributing to resilience and adherence among patients with diabetes. However, the interaction of these variables within the Namibian context remains under-explored, warranting investigation (15, 16) (See **Figure 1**).

By analyzing the interplay between self-management, self-efficacy, social support, and fasting blood glucose levels, this research aims to identify critical predictors and barriers to effective diabetes care (15, 16). Findings from this study are intended to inform targeted intervention programs that leverage the HPM to enhance diabetes education, improve patient self-management capabilities, and ultimately facilitate better health outcomes. It can devise actionable strategies tailored to the unique challenges faced by diabetic patients in Windhoek, contributing to improved healthcare delivery and patient support systems across Namibia.

### A conceptual framework

In our study, the conceptual framework using the HPM was employed. The concept for this study was built based on empirical and theoretical review, which gave the key variables of the study: self-management as an independent variable, fasting blood glucose as a dependent variable, and self-efficacy and social support as moderators. Nola Pender’s HPM explored the probable link between diabetes self-management and fasting blood glucose and the moderating effect of self-efficacy and social support on this relationship. The theory provided a background for collecting and analyzing data in this study (14).

## Materials and methods

### Study design

An analytical cross-sectional study design was conducted using a facility-based, quantitative approach. The study gathered data from many different diabetic patients at a single point in time and gained insight regarding the specified objectives.

### Study setting

The study focused on the Windhoek district in the Khomas region, the only district and the most populous area in Namibia (17). Twelve health facilities in Windhoek feature specialized Non-Communicable Disease (NCD) consultation rooms, including diabetes mellitus. These health facilities are Katutura Health Centre, Khomasdal Health Center, Okuryangava Health Center, Dordabis Clinic, Wanaheda Clinic, Otjomuise Clinic, Hakahana Clinic, Robert Mugabe Clinic, Groot-Aub Clinic, Baumgartsbrun Clinic, Donkerhoek Clinic and Maxuilili Clinic (18). However, the Robert Mugabe Clinic operated as a COVID-19 center during data collection. According to the Ministry of Health and Social Services (MoHSS), these facilities attend to approximately 10,802 DM cases, of which 9721 (90%) are T2DM (18).

### Study participants

We included T2DM patients receiving follow-up care at the selected healthcare facilities. We included individuals aged ≥18 years and diagnosed with T2DM. Participants were required to have received care from selected public healthcare facilities within the Windhoek district on at least two occasions over the past three months, confirming their consistent attendance at the clinic. Additionally, candidates need to have been on diabetes therapy for a minimum of 12 months and must have provided informed consent to participate in the study. Individuals with poor levels of consciousness or dementia, those facing communication difficulties, critically ill (with severe hepatic or renal impairment) were excluded.

### Sample size

A single population proportion formula was used to estimate the required sample size, which determined that 384 diabetic patients were needed for the study. However, after the initial assessment, the study successfully recruited 315 participants.

### Sampling procedures

Simple random sampling was used to select the region for the study from 14 administrative regions in Namibia. The names of the regions were written on separate pieces of paper, folded, and thoroughly mixed. One paper was drawn randomly, revealing the Khomas region as the chosen study area. Six of the 12 health facilities in the region were selected through a lottery method, with each having different NCD consultation rooms, including endocrinology. All six endocrinology consultation rooms were included in the study. A target sample size of 384 participants was evenly distributed across the six health facilities, with 64 participants selected from each. Systematic random sampling was applied to the follow-up entry registers, where every second participant was chosen. If a selected participant did not meet the eligibility criteria, the next person in line was selected. Interviews were conducted five days a week, from 8:00 a.m. to 11:00 a.m., in a convenient and private room. This resulted in 384 randomly selected participants from six endocrinology consultation rooms across six health facilities.

### Study variables

The dependent variable identified in this research was FBG levels, critical glycaemic control indicators in individuals with T2DM. Additionally, social support and self-efficacy were designated as moderators in this study.

Independent variables included demographic characteristics and self-management practices. Demographic characteristics: age in years, gender, education level, and socioeconomic status. The focus on self-management practices encompassed the patients’ ability to actively manage their diabetes through adherence to medication, dietary choices, and lifestyle modifications. Social support refers to the assistance and encouragement provided by family, peers, and healthcare providers, which can enhance the quality of self-management behaviors. Meanwhile, self-efficacy pertains to an individual’s belief in their ability to successfully execute the necessary behaviors to regulate their condition, significantly impacting their overall diabetes management and health outcomes.

### Data collection procedure and measurement tools

Data collection was conducted by trained research assistants. Participants were recruited through a combination of ethical and equitable recruitment methods that included distributing study information via flyers and direct person-to-person contact in settings appropriate for the target population, ensuring the protection of participants’ rights and avoidance of coercion. Recruitment venues were within the hospital clinic offices. Prospective participants were invited to contribute through personalized face-to-face interactions by research staff knowledgeable about the study, who provided clear explanations of the research objectives and procedures. When necessary, interpreters were made available to overcome language barriers and facilitate understanding. After obtaining informed consent, participants were given the questionnaire, which was collected on the same day once completed. The questionnaires were collected and returned in the same day. Trained native-speaking interviewers were present to assist illiterate participants by reading the questions aloud and clarifying any doubts, thus providing essential support during data collection. Data collection took place over four months, from July to October 2023. The questionnaire consisted of 40 questions divided into five sections, covering demographic information, self-management, FBG levels, self-efficacy, and social support.

#### Self-management

To measure self-management, we adapted 11 items from the expanded version of the Summary of Diabetes Self-Care Activities (SDSCA) developed by Toobert et al. with good reliability and validity (19). Adequate self-management was defined as adhering to the daily recommended behaviors of diet, medication, and foot care over the last week and being physically active for at least 30 minutes on three days of the week.

#### Fasting blood glucose

This study used the FBG measure as an alternative to assess the blood glucose levels of T2DM patients. Using the past FBG conducted within the last 2 months extracted from the patient’s medical records, the patient’s glycaemic control status was determined by calculating the mean of three FBG measurements. After an overnight fast, blood glucose levels were assessed using the HemoCue 201+ blood glucose analyzer with capillary whole blood from adults’ middle or ring finger. The finger was cleaned with 70% isopropyl alcohol, pricked with a retractable lancet, and the first drop of blood was discarded. The second drop was drawn into the glucose micro cuvette through capillary action, and the micro cuvette was then placed into the HemoCue 201+ analyzer to obtain a glucose reading in millimoles per liter (mmol/L). For this study, FBG control was considered successful and optimal when the FBG level ranged between 4.0 and 7.0 mmol/L (20, 21).

#### Self-efficacy

Self-efficacy was measured with the Perceived Diabetes Self-Management Scale (PDSMS), an 8-item questionnaire. A word substitution procedure was applied for ‘condition’ with ‘diabetes’, ensuring specificity to the condition. This is a validated, reliable measure of diabetes self-efficacy with a Cronbach alpha of 0.83. The items of the PDSMS elicit responses on a scale of 1 to 5, where 1 indicates “Strongly Disagree”, and 5 represents “Strongly Agree”. These four items (1, 2, 6, & 7) were phrased where strong agreement indicates lower self-efficacy or perceived competence. These four are reverse-scored before being combined with the remaining four items. The overall PDSMS score can vary between 8 and 40, with higher scores reflecting greater confidence in managing one’s diabetes (22).

#### Social support

Social support was measured using a widely used tool adapted from the Medical Outcomes Study. A nine-item scale was utilized to measure social support. It contained a Likert scale ranging from 1 to 5, asking participants to indicate how much support they can count from people around them with 1 – None of the time; 2 – a little of the time; 3 – some of the time; 4 – most of the time and 5 – all of the time. Those indicated most or all of the time are classified as having good support, while those with some of the time and a little of the time as moderate. Social support and the items showed reliability and construct validity, with Cronbach’s alpha coefficient ranging from 0.74 to 0. 93. This instrument evaluated the presence of emotional, informational, and networking support and various sources of social support. The scale included emotional and informational, practical, affectionate support, and positive social interaction (23).

### Data management and analysis

Upon receiving the completed questionnaires, each was assigned a unique identification number. The researcher then entered the data into Microsoft Excel for preliminary organization and double checking before analysis. The data were analyzed using the Statistical Package for Social Sciences (SPSS) version 29. Descriptive statistics were used to summarize continuous variables with means and standard deviations, while categorical variables were summarized by frequency and proportions, and presented in tables and graphs. Pearson correlation analysis was performed to explore the relationship between self-management and FBG levels. Additionally, linear regression and moderation analyses were conducted to assess the moderating effects of self-efficacy and social support on the relationship between self-management and FBG. A p-value of <0.05 was considered statistically significant.

## Results

### Demographic and health-related factors analysis

**Table 1** present demographic and health related characteristics. A total of 315 participants were analyzed, with a slightly higher representation of males (51.4%). Most respondents were aged 40-59, accounting for 58.1% of the sample. Most participants were single (59.4%), and income levels varied, with 36.8% earning below $105 monthly. A significant portion had at least a certificate or higher education (49.5%). Regarding employment, 65.1% were employed, while 16.8% were unemployed. Most individuals reported a diagnosis duration of 1–5 years (46%), with hypertension being the most common comorbidity (43.8%). Among diabetes complications, 85.7% reported no complications, and the majority (85.4%) were non-smokers. (**Table 1**).

**Table 1:** Demographic and health-related factors of study participants (n=315).

Variable	Frequency	Percentage
Gender		
Male	162	51.4
Female	153	48.6
Age group		
18 - 29	21	6.7
30 - 39	67	21.3
40 - 49	89	28.3
50 - 59	94	29.8
60+	44	14
Marital status		
Single	187	59.4
Married	83	26.3
Widowed	33	10.5
Divorced	12	3.8
Income (per month) in US Dollars (US$)		
Below 105	116	36.8
106 – 265	61	19.4
266 - 525	41	13
≥526	97	30.8
Education level		
Illiterate	67	21.3
Up to Primary	34	10.8
Up to high school	58	18.4
Certificate & up	156	49.5
Occupation		
Student	8	2.5
Unemployed	53	16.8
Employed	205	65.1
Retired	49	15.6
Diagnosis duration		
<1 year	35	11.1
1–5 years	145	46
6–10 years	85	27
>10 years	50	15.9
Comorbidities		
Hypertension	138	43.8
Stroke	1	0.3
Kidney failure	7	2.2
Asthma	43	13.7
Epilepsy	3	1
Hyperlipidemia	83	26.3
None	40	12.7
Diabetes complications		
Foot damage	15	4.8
Eye damage	4	1.3
Kidney damage	26	8.3
None	270	85.7
Smoking		
Yes	46	14.6
No	269	85.4

### Self-management practices, FBG, self-efficacy, and social support levels among participants

Regarding self-monitoring of blood glucose, 34.3% of respondents reported doing so, while a majority (65.7%) did not. On average, individuals engaged in dietary practices 3 days per week and foot care 4 days, while exercising more frequently at 5.5 days per week. Medication adherence was notably high, averaging 6.9 days per week. Participants reported smoking an average of 11 cigarettes per day. In self-efficacy levels among participants, results revealed a mean self-efficacy score of 24.31 (standard deviation=2.8). Seven in ten (71.2%) reported low self-efficacy. The mean score of social support was 30.79 (± 9.3), and more than two-thirds (72.3%) reported high social support. (**Table 2**)

**Table 2:** Patients’ self-management practices, self-efficacy, and social support.

Variable	n	%	Mean days/week	SD
Diet			3	1.72
Exercise			5.5	1.94
Foot care			4	2.63
Medication			7	0.56
Average cigarette smoked per day			11	3.84
Self-monitoring of blood glucose				
Yes	108	34.3		
No	207	65.7		
Smoking last 7 days				
Yes	46	14.6		
No	269	85.4		
Self-efficacy			24.31	2.773
Low self-efficacy	210	71.2		
High self-efficacy	105	28.8		
Social support			30.79	9.28
Low social support	101	27.7		
High social support	214	72.3		

SD, Standard deviation.

### FBG levels

**Table 3** presents descriptive statistics summarizing FBG levels among patients with diabetes across three consecutive months: July, August, and September. In July, the mean FBG level was 9.32 mmol/L, with a standard deviation 4.54. The minimum recorded value was 4.1 mmol/L, while the maximum was substantially higher at 10.7 mmol/L, resulting in a wide range of 6.6 mmol/L. (**Table 3**)

**Table 3:** Descriptive statistics for fasting blood glucose across months.

Variable	Mean	SD	Minimum	Maximum	Range
July	9.32	4.54	4.1	10.7	6.6
August	9.19	4.21	4.2	10.4	6.2
September	9.05	4.51	4	10.5	6.5

### Relationship between self-management practices and FBG

The correlation coefficient for the relationship between patients’ self-management practices of diabetes and their FBG is -0.349 (p < 0.028).

### Regression analysis of self-management on the FBG level with moderation in self-efficacy and social support

The regression model’s overall R-squared value was 0.431, indicating that 43.1% of the variation in FBG was explained by the independent factor of self-management and the moderator’s self-efficacy and perceived social support. Self-management emerged as a significant predictor of fasting blood glucose levels, exhibiting a negative coefficient (β = -0.903, 95% CI = -1.046 - -0.756, p < 0.001). Similarly, self-efficacy was also significantly associated with fasting blood glucose levels (β = -0.575, 95% CI = -0.729 - -0.42, p < 0.001). Social support was negatively associated with fasting blood glucose levels (β = -0.485, 95% CI = -0.729 - -0.42, p < 0.001). (**Table 4**)

**Table 4:** Regression analysis of the moderating effects of self-efficacy and social support on the relationship between self-management and FBG.

Variable	β (95% CI)	SE	t	p-value
1	-12.745 (-15.022 - -10.467)	1.157	-11.009	<0.001
Self-management	-0.903 (-1.046 - -0.756)	0.073	-12.384	<0.001
Social support	-0.485 (-0.648 - -0.323)	0.083	-5.868	<0.001
Self-efficacy	-0.575 (-0.729 - -0.42)	0.079	-7.305	<0.001

Adjusted R^2^ = 0.431, F (28.87, p <.001).

### Moderating effect of social support on the relationship between self-management and FBG

The results indicated that social support significantly moderates this relationship. Specifically, at one standard deviation (SD) below the mean of social support (M = 21.338), the effect of self-management on FBG was associated with a decrease in FBG levels for patients with lower levels of social support (B = -0.233, SE = 0.039, t = -6.536, p <.001). At the mean level of social support (M = 25.752), the effect was more substantial that as social support increases to an average level, the association between self-management and lower FBG levels becomes more pronounced (B = -0.339, SE = 0.025, t = -14.074, p <.001). At one standard deviation above the mean (M = 30.166), the effect was even more substantial that high levels of social support greatly enhance the beneficial impact of self-management on FBG levels (B = -0.424, SE = 0.017, t = -26.109, p <.001).

The analysis also explored the moderating effect of self-efficacy on the relationship between self-management and FBG levels. The results showed that self-efficacy significantly moderates this relationship. At one standard deviation below the mean of self-efficacy (M = 21.174), the effect of self-management on FBG was linked to a reduction in FBG levels for patients with lower levels of self-efficacy (B = -0.263, SE = 0.039, t = -5.966, p <.001). At the mean level of self-efficacy (M = 25.676), the effect was more assertive that at average levels of self-efficacy, self-management is more effectively associated with lower FBG levels (B = -0.353, SE = 0.025, t = -13.672, p <.001). At one standard deviation above the mean (M = 30.182), the effect was most pronounced, indicating that high levels of self-efficacy significantly amplify the positive impact of self-management on FBG levels (B = -0.446, SE = 0.017, t = -26.109, p <.001). (**Table 5**).

**Table 5:** Conditional effects of the moderators of self-efficacy and social support on the relationship between self-management and FBG.

Measure	Value	Effect	SE	t	p
Social Support					
Mean - 1 SD	21.338	-0.233	0.039	-6.536	<0.001
Mean	25.752	-0.339	0.025	-14.074	<0.001
Mean + 1 SD	30.166	-0.424	0.017	-26.109	<0.001
Self-efficacy					
Mean - 1 SD	21.174	-0.263	0.039	-5.966	<0.001
Mean	25.676	-0.353	0.025	-13.672	<0.001
Mean + 1 SD	30.182	-0.446	0.017	-26.109	<0.001

**Figure 2:**
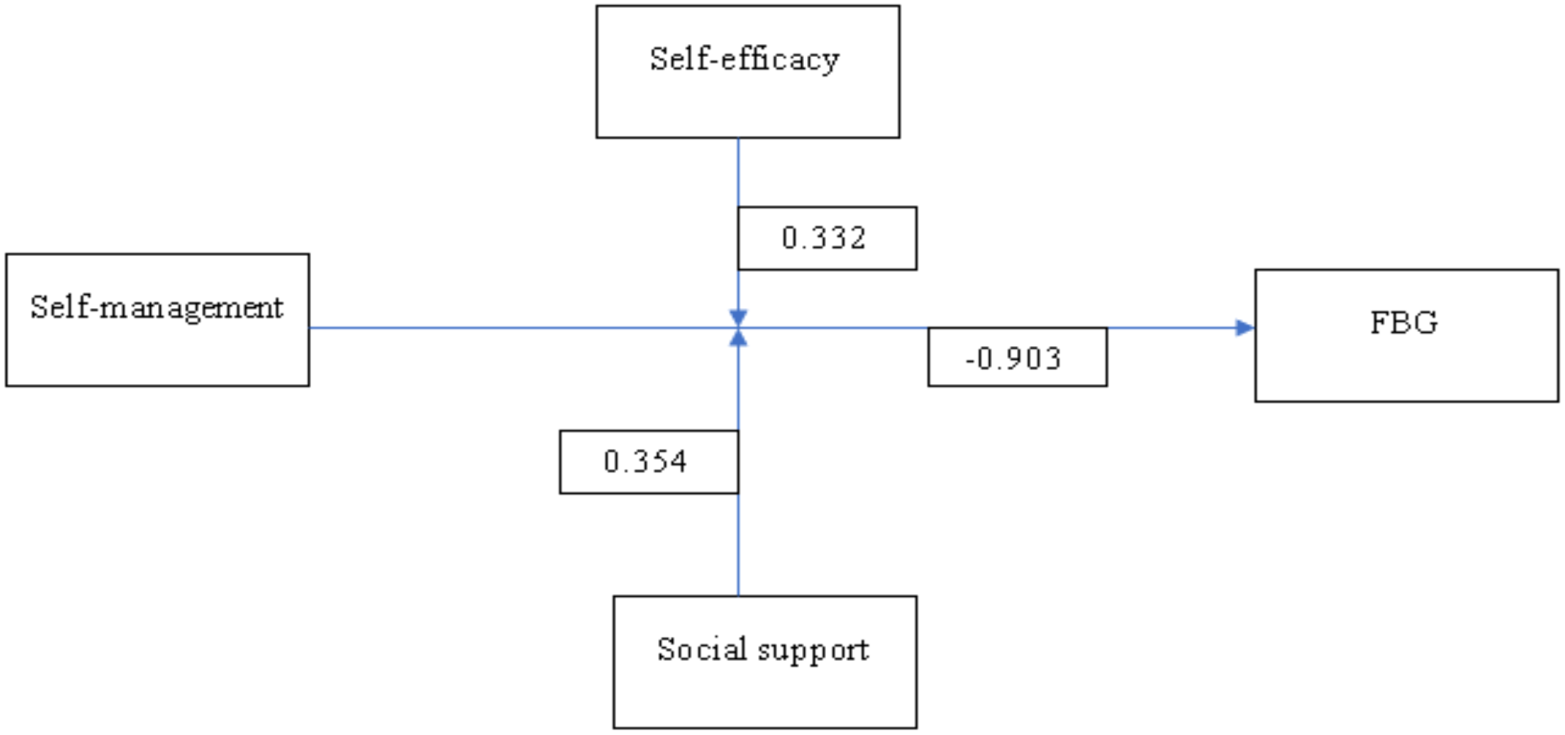
Moderation effects of social support and self-efficacy.

**Figure 2** presents the moderation effects of social support and self-efficacy on the relationship between self-management of diabetes and FBG for patients. The diagram indicates that self-efficacy moderates the relationship between self-management and FBG, as suggested by a coefficient of 0.332. Additionally, the coefficient for the pathway from self-management to social support is 0.354. However, the direct effect of self-management on FBG is notably negative (-0.903), which indicates that, in the absence of moderators, increased self-management alone may lead to lower FBG levels.

## Discussion

We aimed to use the Health Promotion Model to explain the relationship between self-management and fasting blood glucose among T2DM patients in Namibia by also assessing the moderating effects of self-efficacy and social support. The findings regarding self-management practices among participants highlight significant gaps and strengths in diabetes care. Specifically, a few respondents reported self-monitoring blood glucose, which is crucial for effective diabetes management. This low percentage suggests that many individuals may not adequately track their glycemic levels, risking poor metabolic control. However, the study observed relatively better adherence to other self-management practices, such as medication adherence, which was notably high almost daily. This aligns with literature emphasizing the importance of medication compliance in maintaining optimal glycemic control and reducing the risk of complications associated with diabetes (24). Moreover, the frequency of physical exercise (5 days per week) indicates a positive trend in lifestyle behavior modification, as physical activity is associated with improved insulin sensitivity and cardiovascular health in diabetic patients (25). Nevertheless, the average of 10.98 cigarettes smoked daily raises concerns, given that smoking is well-documented to exacerbate diabetes-related complications (26).

The self-efficacy levels observed in this study reveal a crucial understanding of the psychological dimensions of diabetes management. Research indicates that higher self-efficacy is correlated with better self-management practices and glycemic control, underscoring the role of psychological health in chronic disease management (27). In contrast, social support levels were encouraging. This finding is noteworthy as existing literature demonstrates that robust social support networks can enhance self-management behaviors by providing encouragement and resources necessary for effective diabetes care (28, 29). Thus, while certain self-management practices, such as medication adherence, are strong among participants, the low levels of self-efficacy highlight an area for targeted interventions, which could leverage existing social support to foster improved diabetes management outcomes.

The FBG results showed considerable variability in glycemic control among participants, consistent with existing literature highlighting the fluctuating nature of blood glucose levels in individuals with diabetes (30). The recorded values suggest that while some patients may achieve commendable glycemic control, others experience significant hyperglycemia, reflecting the challenges of managing diabetes effectively. The mean FBG findings from participants demonstrate ongoing improvements in managing blood glucose levels, essential for preventing complications associated with poorly regulated diabetes. Despite the positive trend, the persistence of notable range values emphasizes the critical need for continuous monitoring and individualized intervention strategies tailored to patient-specific profiles to optimize glycemic control and improve overall health outcomes.

The findings of this study suggest that as self-management practices improve, FBG levels tend to decrease, although the strength of this association is modest. Existing literature supports this finding, demonstrating that effective self-management behaviors, such as regular blood glucose monitoring, adherence to dietary recommendations, and consistent medication use, are crucial for achieving better glycemic control (31). Furthermore, studies indicate enhanced self-management leads to greater patient empowerment and improved health outcomes (24, 28, 32). Despite the weak correlation, the statistical significance highlights the importance of self-management practices as a vital component in diabetes care. This suggests that interventions to enhance self-management could potentially foster improved metabolic control among patients.

Findings also showed the multifaceted nature of diabetes management as framed by the HPM. Similarly, self-efficacy emerged as a significant predictor, reinforcing that individuals who believe in managing their diabetes are more likely to engage in health-promoting behaviors, which positively affect FBG levels (16, 27). Furthermore, the negative association of social support with FBG levels underscores the vital role of supportive social networks in diabetes self-management; individuals with stronger social support systems are reported to have better adherence to care plans and improved glycemic control (15, 29). ​Together, these findings emphasize the importance of integrating self-management education that enhances self-efficacy and fosters social support to manage diabetes effectively within the HPM framework.

The findings indicate that social support significantly moderates the relationship between self-management practices and FBG levels in patients with diabetes.​ As social support increases, the relationship strengthens considerably, suggesting that higher social support substantially enhances the efficacy of self-management behaviors in reducing FBG levels. This supports the existing literature, consistently showing that strong social support networks contribute to better self-management and health outcomes in diabetic populations (28). Numerous studies confirm that social support can improve adherence to diabetes self-care activities, including dietary management and physical activity, thereby facilitating more effective glucose control (15, 28). Conversely, some literature suggests that excessively supportive environments may hinder independence in managing diabetes, creating dependency rather than empowerment (33). These contrasting findings highlight the need for future research to explore the nuances of social support’s role in diabetes management.

### Strengths and limitations

This study, while contributing valuable insights into self-management and glycemic control in T2DM through a HPM framework, faces several methodological challenges and limitations that warrant consideration. First, the reliance on self-reported data for diabetes self-management behaviors and glycemic control introduces potential biases, including social desirability and recall bias, which may compromise the accuracy of the findings. Additionally, the use of cross-sectional study design limits the ability to infer causal relationships between self-efficacy, social support, and glycemic outcomes, as temporal precedence is not established. Participant characteristics such as literacy levels present challenges; with 21% of the sample being illiterate, variations in health literacy likely influenced data quality and self-management adherence. Furthermore, sample size constraints limit the generalizability of results to the broader population of individuals with T2DM. Cultural factors, which can significantly affect self-management behaviors and perceptions of social support, were not exhaustively explored, potentially overlooking important contextual moderators. The instruments used to measure self-efficacy and social support also entail inconsistencies and possible measurement errors, including social desirability bias and lack of longitudinal validation, which affect the reliability of moderation effect assessments. Lastly, technological or resource constraints restricted the implementation of objective self-management measures, relying predominantly on subjective reports that may not capture the full complexity of diabetes management behaviors. Lastly, factors such as comorbidities and psychological conditions were not controlled for, which could further confound the relationships explored in this research.

### Implications

The HPM offers a valuable framework for understanding the moderating effects of both social support and self-efficacy on the self-management-FBG relationship. The model emphasizes that perceived benefits and interpersonal influences, such as social support, are crucial in shaping behaviors that promote health. It posits that when patients perceive high levels of social support, their self-efficacy may increase, leading to more consistent engagement in healthy self-management behaviors. As demonstrated in the findings, self-efficacy also serves as a significant moderator, where increasing levels substantially enhance the positive effect of self-management on FBG levels. The literature corroborates this, revealing that higher self-efficacy is linked to improved self-care, adherence, and better glycemic control (16, 27, 28, 31). However, discrepancies arise in studies that observe the overwhelming influence of external factors, such as diabetes-related conflicts, which may overshadow self-efficacy benefits (6, 13). The health promotion model can guide future interventions by integrating strategies to improve social support and self-efficacy tailoring educational programs that foster a supportive environment while enhancing patients’ confidence in managing their diabetes effectively.

## Conclusion

In conclusion, this study revealed the significant moderating roles of social support and self-efficacy in the relationship between self-management practices and fasting blood glucose levels in patients with diabetes.​ Effective self-management is associated with lower FBG, particularly when patients have high social support and self-efficacy levels. These results highlight the importance of comprehensive diabetes management programs that focus on individual behavioral changes, enhance social support networks, and boost self-efficacy. Further research is needed to explore the causal relationships and underlying mechanisms connecting these variables to inform more effective interventions in Namibia populations and SSA.

## Data Availability

Publicly available datasets were analyzed in this study. The data can be accessed to corresponding author.
